# Physical Activity as a Preventive Lifestyle Intervention Acts Through Specific Exosomal miRNA Species—Evidence From Human Short- and Long-Term Pilot Studies

**DOI:** 10.3389/fphys.2021.658218

**Published:** 2021-08-02

**Authors:** Kitti Garai, Zoltan Adam, Robert Herczeg, Krisztina Banfai, Adam Gyebrovszki, Attila Gyenesei, Judit E. Pongracz, Marta Wilhelm, Krisztian Kvell

**Affiliations:** ^1^Department of Pharmaceutical Biotechnology, Faculty of Pharmacy, University of Pécs, Pécs, Hungary; ^2^Wnt-Signaling Research Group, Szentagothai Research Center, University of Pécs, Pécs, Hungary; ^3^Bioinformatics Research Group, Szentagothai Research Center, University of Pécs, Pécs, Hungary; ^4^Faculty of Science, Institute of Sport Sciences and Physical Education, University of Pécs, Pécs, Hungary

**Keywords:** regular exercise, exosome, miRNA, chronic disease, prevention

## Abstract

Exercise initiates systemic adaptation to promote health and prevent various lifestyle-related chronic diseases. Emerging evidence suggests that circulating exosomes mediate some of the beneficial effects of exercise via the transfer of microRNAs between tissues. Yet to date, a comprehensive profile of the exosomal miRNA (exomiR) content released following short-term (0.5 year in this study) and long-term (25 + years in this study) regular bouts of exercise is still lacking. However, a better understanding of these miRNA species would assist in clarifying the role of regular exercise at the molecular level in the prevention of chronic diseases. In the present pilot studies we analyzed serum exomiR expression in healthy young, sedentary participants (*n* = 14; age: 23 ± 2 years) at baseline and following a half year-long moderate-intensity regular exercise training. We also analyzed serum exomiR expression in older, healthy trained participants (seniors, *n* = 11; age: 62 ± 6 years) who engaged in endurance activities for at least 25 years. Following the isolation and enrichment of serum exosomes using Total Exosome Isolation Reagent (TEI) their exomiR levels were determined using the amplification-free Nanostring platform. Hierarchical cluster analysis revealed that the majority of exomiRs overlap for short-term (0.5 year in this study) and long-term (25 + years in this study) regular bouts of exercise. The top 12 significantly altered exomiRs (let-7a-5p; let-7g-5p; miR-130a-3p; miR-142-3p; miR-150-5p; miR-15a-5p; miR-15b-5p; miR-199a-3p; miR-199b-3p; miR-223-3p; miR-23a-3p, and miR-451a-3p) were used for further evaluation. According to KEGG pathway analysis a large portion of the exomiRs target chronic diseases including cancer, neurodegenerative and metabolic diseases, and viral infections. Our results provide evidence that exosomal miRNA modulation is the molecular mechanism through which regular exercise prevents various chronic diseases. The possibility of using such exomiRs to target diseases is of great interest. While further validation is needed, our comprehensive exomiR study presents, for the first time, the disease-preventive molecular pattern of both short and long-term regular exercise.

## Introduction

Regular exercise has been known as a major intervention tool not only to attenuate the risk of a multitude of disorders from metabolic disease and neurodegenerative disorders to cancer, but also to delay the occurrence of numerous age-related diseases (Brahmer et al., 2019). While most molecular mechanisms mediating the long-term beneficial effects of exercise remain unexplored, growing evidence suggests the involvement of tissue crosstalk via the release of exosomes following exercise (Frühbeis et al., 2015; Estébanez et al., 2021). Exosomes are small extracellular vesicles (sEVs) (30–150 nm) that are secreted by fusion of multivesicular bodies to the plasma membrane (Brennan et al., 2020). These vesicles transport a large variety of cargo molecules including miRNAs, DNA and proteins that may be taken up by distant cell types and alter the phenotype of these recipients (Kowal et al., 2014). Since miRNA species are well recognized for playing important roles in many physiological and pathological processes, they could also be involved in exercise-related benefits of disease prevention. Deciphering the contribution of miRNAs present in exercise-derived exosomes and their downstream targets is crucial for the better comprehension of how preventive lifestyle actually acts at the molecular level. According to a study, the expression of certain circulating miRNA species increases with age in plasma microvesicles (Rani et al., 2017). Of notable example, hsa-miR-223-3p, hsa-miR-23a-3p, hsa-let-7g-5p, hsa-miR-199a-5p, hsa-miR-15a-5p, and has-miR-142-3p show positive correlation with age and the development of specific chronic diseases (Rani et al., 2017). Recently it has been shown that healthy aging is also reflected by the profile of circulating exosomes, and exercise−induced beneficial effects may be related with the modulation of these exosomes (Bertoldi et al., 2018). There are reports indicating the changes of various miRNA species in exosomes following an acute of exercise (D’souza et al., 2018; Yin et al., 2019), however only a small number of studies examine exosomes in response to long-term training (Nederveen et al., 2021). Of note, in a mammalian study the levels of miR−19b, miR−148a, miR−150, miR−221, miR−361, and miR−486 were up-regulated during the first month of exercise, but returned to baseline by completion of a 4−month study period (Muroya et al., 2015). Regarding long-term human experiments, a significant increase in exosome release was shown after a single bout of flywheel exercise (Annibalini et al., 2019), whereas no change was found after a full year of rowing training (Hou et al., 2019). These conflicting results could potentially be attributed to the adaptation process that occurs with time. Additional research is crucial with various training modalities and durations to further understand the role of exosomes and their miRNA content in the prevention of chronic diseases induced by long-term exercise. In the present study first we investigated the effect of a 0.5 year-long, moderate intensity, personal trainer-supervised, concurrent resistance and aerobic training program on the overall circulating exomiRs expression profile of healthy, young, previously sedentary individuals. We also assessed whether exomiRs differentially expressed after a 0.5 year regular exercise in young adults were similarly present in healthy senior trained participants who have engaged in regular exercise activities for at least 25 years. The effect of short- and long-term regular exercise on the miRNA profile was determined by comparing baseline vs. 0.5 year, and baseline vs. 25 + years miRNA levels. As anticipated we found that the levels of the exomiRs are fairly consistent in comparison of the 0.5 year (short-term adaptation) and the 25 + years (long-term adaptation) active groups. Twelve exomiRs showed overlap for both study periods (baseline vs. 0.5 year and baseline vs. 25 + years). Of note, all of them were significantly down-regulated. Bioinformatics analysis was used to evaluate the interplay between biological signaling pathways offering insight into mechanisms linking exercise and chronic disease prevention. Our results prove that full miRNome analysis might be a useful tool to identify exomiRs acting on particular pathways that prevent the development of specific chronic diseases.

## Materials and Methods

### Participants and Applied Training Protocol

Healthy young, sedentary (*n* = 14; age: 23 ± 2 years) and senior trained (*n* = 11; age: 62 ± 6 years) individuals were recruited. Participants were in good general health, defined as having no chronic diseases (e.g., metabolic disorders, cardiovascular disease, cancer, etc.). Main characteristics of the subjects are summarized in Table 1 (see Supplementary Material for further details). Healthy, young sedentary individuals completed moderate-intensity, concurrent resistance and aerobic exercises regular exercise training three times a week for half a year (Garai et al., 2019). Our exercise bouts consisted of four parts: warm-up, resistance training, aerobic exercises and cool-down with stretching. The heart rate of the participants was measured continuously during exercise with a heart rate monitor (Polar Team System, Polar Electro, Finland). Age-predicted maximum heart rates were estimated with the following calculation: 220—age (years). Every trainings began with standardized, active warm-up protocol applying mobility and stability exercises, gymnastic exercises and moderate stretching. After warming-up resistance training was performed. During this part the heart rate of the subjects was allowed to reach 85% of individual heart rate maximum. Aerobic exercises included walking and jogging, if the subject’s heart rate was lower than 65% of the individual heart rate maximum. The cool-down protocol included 2 min of slow walking and 8 min of static stretching exercises of all major muscle groups. Participants were asked to keep their diet and daily activity level unchanged during the 6 month-long lifestyle program. Training diary was prepared during the 6 months and compliance was calculated accordingly.

**TABLE 1:** Subject characteristics.

	**Baseline**	**0.5 years**	***p***	**25 + years**
Age (years)	23 ± 2	23.5 ± 2		62 ± 6
BMI	21.64 ± 1.57	21.46 ± 1.44	0.382	27.92 ± 2.95
Body weight (kg)	60.39 ± 5.42	59.55 ± 5.74	0.166	75.16 ± 7.18
Body fat percentage (%)	31.79 ± 3.39	31.49 ± 3.47	0.61	21.23 ± 6.03
VO_2_ max (ml/kg/min)	36.41 ± 6.67	39.81 ± 6.20*	0.047	32.9 ± 6.99
LDL (mmol/L)	2.35 ± 0.9	2.44 ± 0.83	0.481	3.63 ± 1.19
HDL (mmol/L)	1.81 ± 0.55	2.13 ± 0.61*	0.002	1.68 ± 0.53
Glucose (mmol/L)	4.94 ± 0.39	4.63 ± 0.31**	< 0.001	5.67 ± 0.45
Systolic BP (Hgmm)	114.5 ± 14.18	108.07 ± 8.69	0.55	131.2 ± 19.42
Diastolic BP (Hgmm)	76.07 ± 9.19	72.93 ± 7.92	0.223	87.00 ± 9.25

Values are expressed as mean ± SD. Paired t-test (*p < 0.05; **p < 0.001); p-values were calculated for baseline vs. 0.5 year as applicable; VO_2_ max, maximal oxygen uptake (cardiorespiratory fitness); LDL, Low Density Lipoprotein; HDL, High Density Lipoprotein; BP, Blood Pressure.

Senior trained subjects were engaged in regular exercise activities for at least 25 years. The exercise behavior of senior participants was assessed with the use of a general lifestyle questionnaire as well as with the International Physical Activity Questionnaire (IPAQ) (Craig et al., 2003). We obtained information on smoking-, alcohol consumption status and physical activity (frequency, type, duration). Senior trained participants also performed both types of exercise (endurance and resistance training), including running, swimming, weightlifting, cycling, skating, adrenaline sports, walking, hiking and spinning. Over half (54%) of the senior participants performed physical activity on a daily basis, while the rest performed physical activity at least twice a week. For details please check the Supplementary Material Section. Each participant gave written informed consent before completing any data collection. The study was conducted according to the Declaration of Helsinki principles and approved by the Regional and Local Ethics Committee of Clinical Center, University of Pecs (ref. no.: 6439/2016 and 7755/2019).

### Collection and Preparation of Human Serum Samples

Human blood samples were collected around 7:00 a.m. after a 12 h fasting in blood collection tubes (BD Vacutainer, SST: BD SST Tubes with Silica Clot Activator and Polymer Gel, Franklin Lakes, NJ, United States) at two time-points: at baseline and after the 0.5 year long training program from the young individuals and at one time-point from seniors. Participants were asked to avoid excessive exercise the day before each testing condition. Blood samples were clotted for approximately 30 min at room temperature. Samples were then centrifuged at 1,500 g for 10 min at room temperature. Serum samples were stored at −80°C until further analysis. The same procedure was carried out with the samples of seniors.

### Exosome Isolation

**FIGURE 1:**
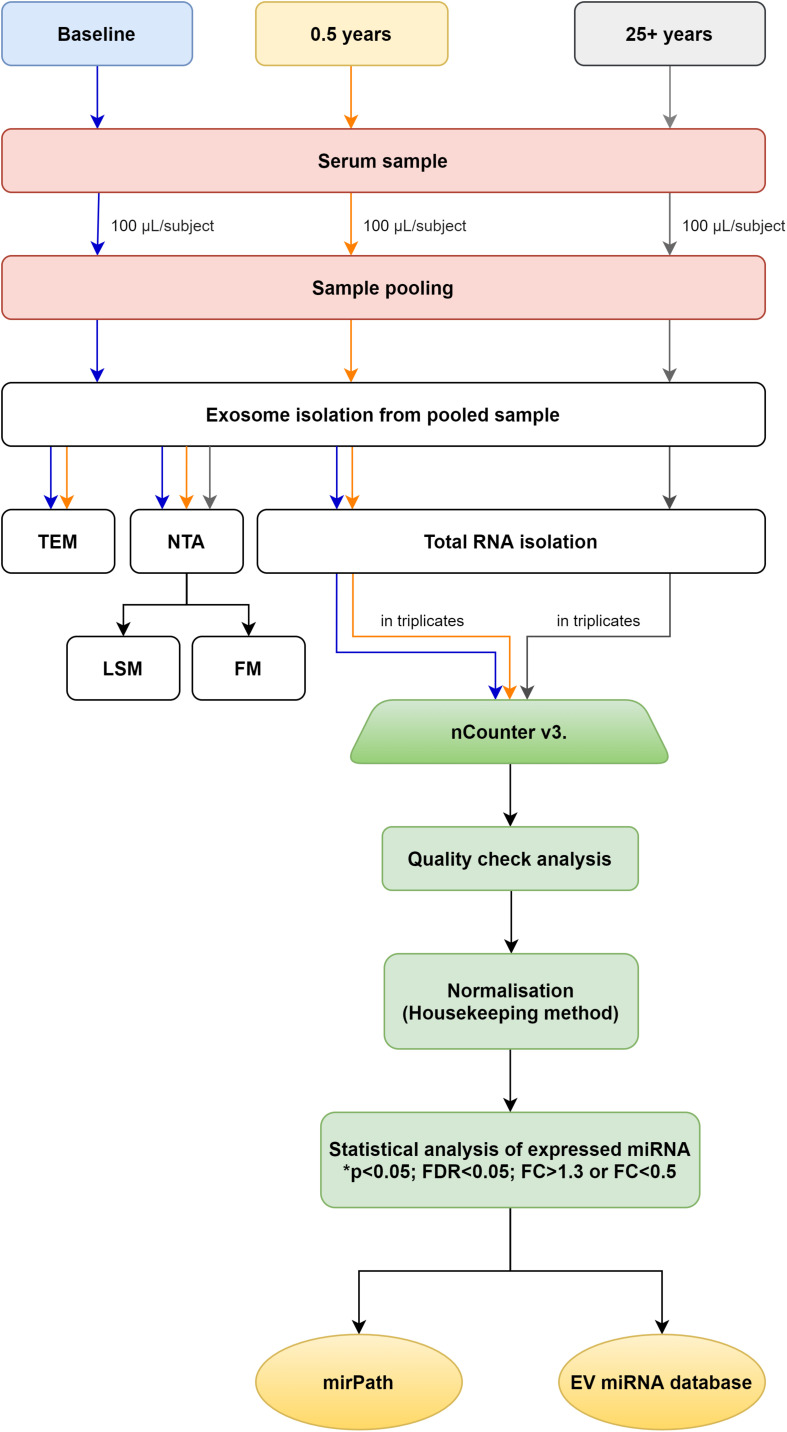
Workflow. Flowchart representing the entire workflow.

In order for their samples to be processed participants had to show min. 85% compliance with regular exercise mandated by the program. Before exosome isolation, equal volumes of serum (100 μl each) from 14 healthy young participants and 11 seniors were pooled, separately (Figure 1). Prior to pooling we have carefully evaluated the participants for potential outliers based on the assessment of physiological and blood parameters. Only those participants’ samples were pooled who constituted a homogenous population for the evaluated physiological and blood parameters (baseline, 0.5 year, 25 + years). Then, exosomes were isolated from the three pooled serum samples (baseline; *n* = 1, 0.5 year; *n* = 1 and 25 + years; *n* = 1 pooled samples) using TEI (from serum) (Invitrogen, Thermo Fisher Scientific, Waltham, MA, United States) following the manufacturer’s protocol. TEI reagents contain volume-excluding polymers (e.g., polyethylene glycol, dextrans, or polyvinyls). According to Andreu et al. (2016) and Banfai et al. (2019), the use of precipitation reagents provide good reproducibility and are suitable for an easy and cost-efficient enrichment of serum exosomes for miRNA analyses. As a result TEI was chosen for studying exosomal miRNA content in our study.

### NTA Measurement With Nanosight NS300

#### NTA Protocol

Exosome-enriched preparations were measured and quantified using Nanosight NS300 instrument (Malvern Panalytical Ltd., Malvern, United Kingdom). The camera level for each sample was manually adjusted to achieve optimal visualization of particles following the manufacturer’s instructions. Samples were injected with a syringe pump (infuse = 50). Detection threshold was set for maximum sensitivity with a minimum of background noise. All measurements were performed in five replicates for each sample, collecting 60-s videos. Following capture, the videos were analyzed by the in-built NTA v3.2 software (Gardiner et al., 2013).

#### Particle Size and Concentration Analysis

The samples of 3 individuals were randomly chosen from each group (baseline, *n* = 3; 0.5 year, *n* = 3; and 25 + years, *n* = 3). All samples were diluted in PBS. Ideal measurement concentrations were achieved by pre-testing the ideal particle per frame value (40–100 particles/frame).

#### Single EV Direct Immunolabeling and NTA Evaluation

The following monoclonal antibodies were used for immune-labeling: anti-human-CD63-FITC (MEM-259) (Thermo Fisher Scientific) anti-human-CD81-PE/Cy7 (TAPA-1) (Sony Biotechnology). Particle concentrations were established for unlabeled EV sample prior to immune-labeling. The concentration of the exosome stock solution was 3.17 × 10^10^ particles/ml (based on NTA). Sample aliquots were prepared to ensure equal dilution effects for each arm of the experiment. Varying concentration anti-CD63 and anti-CD81 antibodies was added to the 50 μl exosome stock solution to yield a volume of 100 μl and to determine an optimized antibody to exosome ratio for single-vesicle labeling. The samples (anti-CD63 labeled; *n* = 1 and anti-CD81 labeled; *n* = 1) were incubated in the dark for 1 h at room temperature. In order to minimize photobleaching during fluorescence mode (FM), all immune-labeled samples were evaluated first in FM, followed immediately by evaluation in light scatter mode (LSM). Then, the FM/LSM percentage was calculated (Thane et al., 2019).

### Transmission Electron Microscopy (TEM)

Exosomes were visualized by transmission electron microscopy. Sample volume of 2.5 μl was placed onto a 300 mesh grid. The grid was left to air dry overnight. Then 5% uranyl-acetate and later 3% sodium-citrate were added to the grid. After 5 min incubation, the grid was allowed to air dry. Twenty four hours later the grid was analyzed using JEOL TEM 1,200 EX. The average diameter of the isolated exosomes was determined using three independent TEM preparations and ImageJ software.

### Exosomal Total RNA Purification and Complete miRNome Profiling

Total RNA from exosomes was extracted using Total Exosome RNA and Protein Isolation Kit (Invitrogen, Thermo Fisher Scientific, Waltham, MA, United States) according to the manufacturer’s protocol. ExomiR level profiling was performed using the Nanostring platform (NanoString Technologies, Seattle, WA) according to the manufacturer’s instructions to analyze 800 human miRNAs. Since the extraction of exosomal miRNA yields low amounts of RNA, but amplification-free methods require high amounts, we adopted the standard method of using pooled samples to yield reproducible reads. Three technical replicates were run per sample (baseline; 0.5 year and 25 + years groups). Quality check confirmed the reliability of the run and also the validity and reproducibility of the miRNA screening protocol. nSolver software was used for data analysis and normalization. Normalization was performed using the Housekeeping method according to nCounter miRNA expression analysis in plasma and serum samples technote instructions. Briefly, NormFinder was used to identify putative housekeeping miRNAs. First, raw data (RCC files) were imported into nSolver and any sample which failed QC was removed. An experiment was built and background subtraction was set to the Mean + 1 SD of the NEG control probes. Of note, we kept normalization options turned off during this process. Data from the completed experiment were exported into an excel file derived from the normalized dataset. Using NormFinder background subtracted data were sorted by average counts across all samples, and all miRNAs expressed below 50 mean counts were deleted when averaged across all samples. NormFinder created a worksheet listing all the genes and a stability value for each of them. With the aid of NormFinder the potential housekeepers with the most stringent stability values were identified. After that we have applied normalization using the geometric mean of five stably expressed miRNAs (hsa-miR-495-3p; hsa-miR-302d-3p; hsa-miR-3144-3p; hsa-miR-612; hsa-miR-548ar-5p) (Andersen et al., 2004). Quality Control fulfilled all the requirements set by the manufacturer.

### Statistical Analysis

All statistical analyses were performed with R (R Core Team, 2019). Paired *t*-tests (baseline vs. 0.5 year) and *t*-tests (baseline vs. 25 + years; 0.5 year vs. 25 + years) were used. We adjusted the *P*-values due to the multiple comparisons therefore False Discovery Rate (FDR) correction was also applied. Heatmap was created in R with the help of “heatmap.2” function from g-plots package (Bolker et al., 2020).

### miRNA Target Prediction and Pathway Analysis

After identifying a dozen similarly expressed exomiRs in the active young and senior groups, miRNA—mRNA signaling pathway interaction analysis was performed. Briefly, online available software mirPath v.3 was used for this purpose (Vlachos et al., 2015). Human database of the mirPath v.3 and the TarBase v7.0 were used for mRNA target prediction. *P*-value and MicroT thresholds were kept as default, 0.05 and 0.8, respectively. False discovery rate (FDR) correction was applied.

### ExomiRs as Biomarkers of Chronic Disease

The exomiR biomarkers related to certain types of chronic diseases were screened through the EVmiRNA database^1^ (Liu et al., 2019). Studies were included if they were original research and evaluated the exomiR levels in a specific disease.

## Results

### Anthropometric and Physiological Parameters

The study comprised 14 healthy, young, previously inactive and 11 senior trained participants. Healthy, young sedentary individuals completed moderate-intensity regular exercise training three times a week for half a year. Senior subjects have done regular exercise for at least 25 years. Participant parameters are listed in Table 1. After half a year of regular exercise, the previously inactive young individuals showed significant improvement in cardiorespiratory fitness (VO_2_max), glucose and lipid metabolism. All physiological parameters of senior trained participants were within a normal range. For them, the VO_2_max values were far better than the age-matched reference range (Supplementary Material).

### Validation of Isolated Exosomes

**FIGURE 2:**
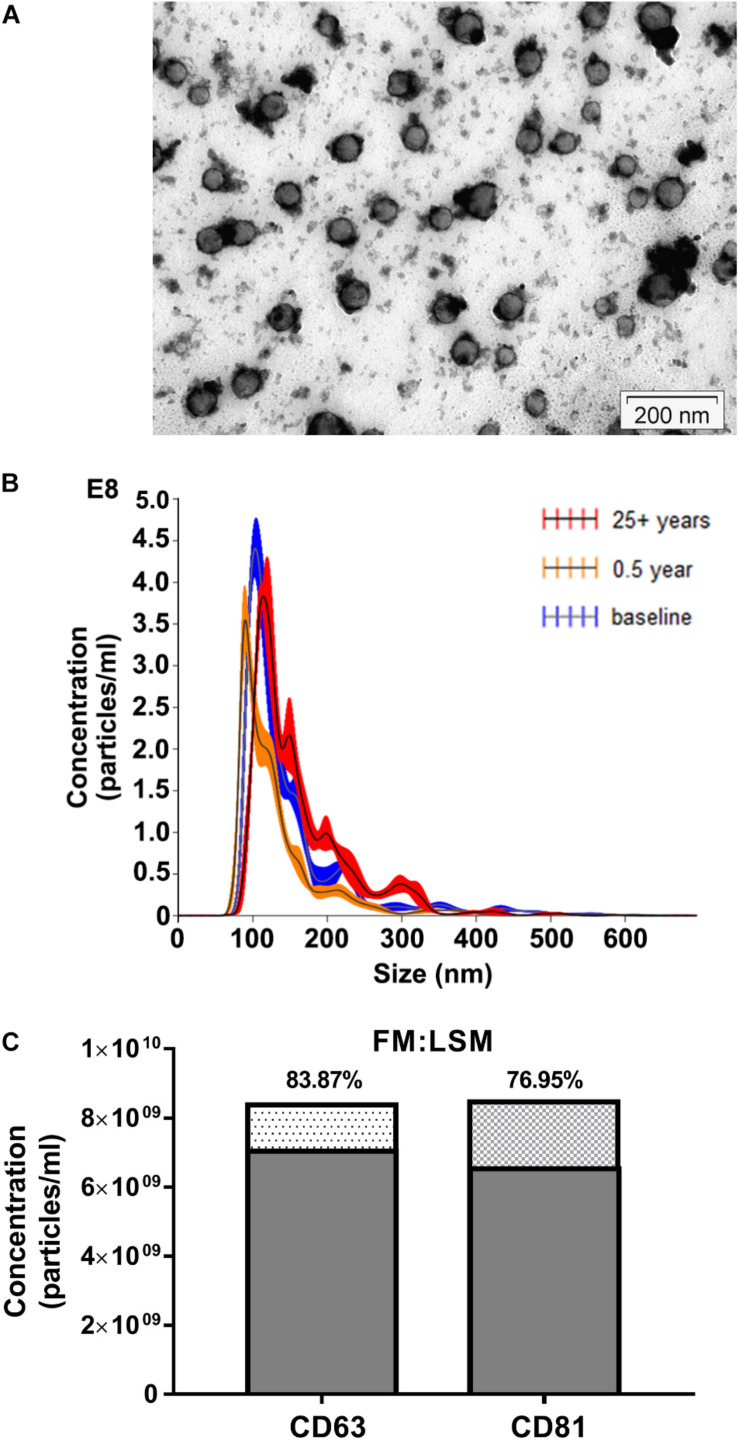
Characterization of exosome-enriched preparations isolated from human serum. **(A)** Transmission electron microscope (TEM) image of exosomes purified from human serum samples (Scale bar: 200 nm). **(B)** Analysis of EV size distribution and concentration in exosome-enriched preparations using a NanoSight NS300 instrument. **(C)** Evaluation of CD63 and CD81 surface expression abundance in exosome-enriched preparations using a NanoSight NS300 instrument.

Exosomes were isolated from blood serum samples and obtained from study participants, as described in section “Materials and Methods.” The purified exosomes were characterized using TEM, a gold-standard technique for nanoparticle validation (Kestens et al., 2017). Our TEM analysis showed typical exosomal round morphology (Figure 2A). Nanoparticle Tracking Analysis (NTA) allowed us to obtain the size distribution of EVs and estimate particle concentration. The mean size of particles (*n* = 9) was 143.2 ± 16.43 nm, which falls into the size range of exosomes (Brennan et al., 2020), confirming that the purified EVs contained exosomes (Figure 2B; see Supplementary Material for further details). Exosome concentrations in our preparations (*n* = 9) ranged from 1.97 × 10^10^ to 3.75 × 10^10^ particles/ml. For details please see the Supplementary Material section. Immune-labeled EV sample was evaluated using NTA in FM and LSM modes. The FM:LSM percentage was 83.87% for of CD63. With the CD81-labeled sample, the FM:LSM percentage was 76.95% (Figure 2C; please also refer Supplementary Videos).

### The Expression Patterns of exomiRs After 0.5 Year of Regular Exercise

**FIGURE 3:**
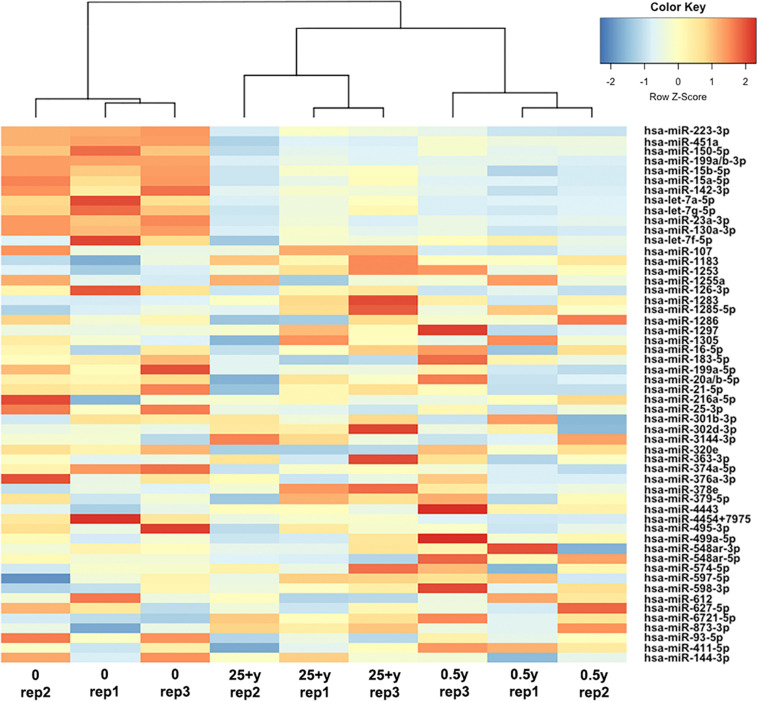
Differential expression of circulating exomiRs. Heatmap with dendrogram shows clustering results for 54 exomiRs at baseline (0), after 0.5 year and 25 + years of regular exercise. Colors represent the level of exomiR expression; red: high expression; blue: low expression.

In order to study regular exercise-related changes in serum exomiR expression, we used amplification-free Nanostring technology. The effect of regular exercise on circulating exomiRs was assessed by comparing baseline (inactive status) and active status (after 0.5 year of regular exercise) expression levels. After analyzing and normalizing raw data, we identified 54 exomiRs (Figure 3). Then, we applied filtering criteria to differentiate baseline vs. 0.5 year results (^∗^*p* < 0.05; #FDR < 0.05; FC > 1.3 or FC < 0.5). Through this analysis, we have observed significant differences in exomiR abundance for several exomiRs (let-7a-5p, *p* < 0.05; let-7g-5p, *p* < 0.05; miR-130a-3p, FDR < 0.05; miR-142-3p, *p* < 0.05; miR-150-5p, *p* < 0.05; miR-15a-5p, *p* < 0.05; miR-15b-5p, FDR < 0.05; miR-199a-3p, FDR < 0.05; miR-199b-3p, FDR < 0.05; miR-223-3p, FDR < 0.05; miR-23a-3p, FDR < 0.05; miR-451a-3p, FDR < 0.05; miR-126-3p, *p* < 0.05; miR-199a-5p, *p* < 0.05; miR-21-5p, FDR < 0.05; miR-25-3p, *p* < 0.05; miR-374a-5p, *p* < 0.05) (for further details please refer to Supplementary Material) (ArrayExpress accession number: **E-MTAB-10067)**.

### ExomiR Overlap of the 0.5 Year- and the 25 + Years of Exercise Groups

Going further we then wished to assess whether exomiRs differentially expressed after 0.5 year of regular exercise were similarly expressed in healthy senior trained participants who engaged in endurance activities for at least 25 years. Therefore, using Nanostring technology we examined the miRNA copy numbers in 11 trained senior individuals focusing on the levels of serum exomiRs. Then, we utilized a hierarchical clustering method to compare circulating exomiR profiles at baseline, after 0.5 year and 25 + years of exercise. As shown by Figure 3, the 0.5 year and 25 + years group share an exomiR expression profile that is completely different from that of the sedentary group. In contrast, the 0.5 year and 25 + years active groups showed a highly similar exomiR expression pattern. In addition, 12 exomiRs (let-7a-5p; let-7g-5p; miR-130a-3p; miR-142-3p; miR-150-5p; miR-15a-5p; miR-15b-5p; miR-199a-3p; miR-199b-3p; miR-223-3p; miR-23a-3p, and miR-451a-3p) showed overlap between the two tested signatures (baseline vs. 0.5 year and baseline vs. 25 + years) (Supplementary Material). Notably, all 12 exomiRs were significantly down-regulated both in the 0.5 year and the 25 + years trained groups as compared to the sedentary group. Having performed a detailed comparison of the 0.5 year vs. 25 + years trained group profiles, miR-411-5p (*p* < 0.05; FC = 0.879) and miR-144-3p (FC = 1.322) showed remarkably different expression. Specifically, miR-411-5p was significantly down-regulated, while miR-144-3p was up-regulated in the 25 + years trained group.

### Pathway Analysis

**FIGURE 4:**
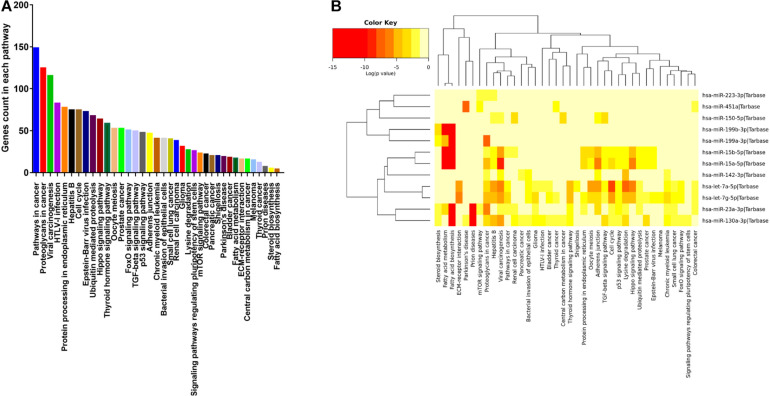
Pathways targeted by the 12 similarly expressed exomiRs in the active young and active senior group. **(A)** Number of genes targeted within each pathways ranked based on high to low. **(B)** The 12 similarly expressed exomiRs in the active young and active senior group regulate 38 signaling pathways in total.

To better understand how these exomiRs may contribute to the health benefits of exercise, we examined the mRNA targets of the 12 similarly expressed exomiRs of the 0.5 year and 25 + year trained groups. Then, to reveal the top targeted pathways associated with each exomiRs, KEGG database analysis was used. We found that 38 KEGG signaling pathways were significantly affected by the 12 selected exomiRs (Figure 4B). Of these Pathways in cancer (hsa05200) had the largest number of targeted mRNAs (148 genes) (Figure 4A). The 148 genes were targeted by four differentially expressed exomiRs (let-7a-5p; let-7g-5p; miR-15b-5p; miR-23a-3p). These findings are consistent with the fact that regular exercise is associated with reduced risk of cancer development. Going further, most exomiRs targeted proteoglycans in cancer pathways (nine exomiRs) and let-7g-5p appeared to affect the most pathways (26 pathways) (for details see Supplementary Material 2).

### ExomiRs as Biomarkers of Chronic Diseases

exomiR biomarkers related to specific diseases were evaluated using an EVmiRNA database. It is the first concise database focusing on miRNA expression profiles in EVs (Liu et al., 2019). Several studies have reported the aberrant expression of the 12 identified exomiRs in various chronic diseases (as summarized by Table 2).

**TABLE 2:** Summary of exomiR biomarkers related to certain diseases according to EVmiRNA database.

**miRNA species**	**Affected age-related chronic disease, autoimmune condition or infection**	**References**
hsa-let-7a-5p	Colorectal-, renal-, prostate-, ovarian-, breast-, lung-, pancreas-, gastric-, esophageal-, thyriod cancer, Ewing’s and Kaposi’s sarcoma, glioblastoma, AML and MML; metastasis formation; cell cycle control; inflammation; diabetes; cardiovascular disease; hepatitis B infection	Iliopoulos et al., 2009; Trang et al., 2010; Lee et al., 2011
hsa-let-7g-5p	Breast-, esophageal-, lung cancer, glioblastoma, AML and CML; graft-vs.-host disease; inflammation; autoimmune thyroid disease; cell cycle control; diabetes; cardiovascular disease; metabolic syndrome; hepatitis B and influenza A infection	Arora et al., 2011; Wang et al., 2013; Biamonte et al., 2019
hsa-miR-130a-3p	Lung-, liver-, prostate-, ovarian-, breast-, cervical-, nasopharyngeal-, prostate cancer, myeloma, CML and glioblastoma; cardiovascular disease; fibrosis; inflammation; autophagy; diabetes; Crohn’s disease; hepatitis C infection; cardiac arrhythmia; renal GBM disease; UV damage	Osbourne et al., 2014; Huang et al., 2015; Eichelmann et al., 2018
hsa-miR-142-3p	Liver-, lung-, colorectal-, breast-, cervical-, esophageal cancer, osteosarcoma, prolactinoma, ALL, AML, CLL and MALT lymphoma; graft rejection; Hashimoto’s thyroiditis; multiple sclerosis; cardiovascular disease; inflammation; rotavirus infection; Alzheimer’s disease; fibrosis	Ma et al., 2016; Sukma Dewi et al., 2017; Wang et al., 2017
hsa-miR-150-5p	Colorectal-, lung-, liver-, prostate-, cervical-, pancreas-, breast-, ovarian-, esophageal cancer, osteosarcoma, glioblastoma, melanoma; Burkitt lymphoma, ALL and MML; inflammation; cardiovascular disease; fibrosis; irritable bowel syndrome; myasthenia; diabetes; SLE; psoriasis	Roderburg et al., 2013; Qu et al., 2014; Yu et al., 2015
hsa-miR-15a-5p	Gastric-, colorectal-, lung-, breast-, liver-, ovarian-, prostate cancer, melanoma, osteosarcoma, neuroblastoma, pheochromocytoma, AML, CLL and multiple myeloma; inflammation; cell cycle control; apoptosis induction; autophagy; multiple sclerosis; hepatitis B infection; fibrosis; diabetes	Xia et al., 2008; Bandi et al., 2009; Sun et al., 2013
hsa-miR-15b-5p	Liver-, gastric-, lung-, liver-, pancreas-, ovarian-, squamous cell cancer, glioblastoma, melanoma, CLL and thymoma; apoptosis induction; metastasis formation; angiogenesis; fibrosis; bipolar disorder; insulin-resistance; skin photoaging; multiple sclerosis; diabetes	Zhang et al., 2015; Li et al., 2016; MacLean et al., 2016
hsa-miR-199a/b-3p	Liver-, gastric-, lung-, renal cell-, ovarian-, pancreas-, colorectal, liver-, breast-, testicular germ cell-, thyroid-, colorectal cancer, endometriosis, glioblastoma, CLL, melanoma, chondrosarcoma and osteosarcoma; osteoarthritis; COPD; autophagy; angiogenesis; HCV infection; inflammation	Li et al., 2015
hsa-miR-223-3p	Ovarian-, gastric-, colorectal-, prostate-, pancreas-, lung-, liver cancer, CLL, AML, ALL, glioblastoma and osteosarcoma; metastasis formation graft rejection; inflammation; osteoarthritis; lipid metabolism; obesity; rheumatoid arthritis; psoriasis; cardiovascular disease; diabetes; COPD; Alzheimer’s disease	Wong et al., 2008; Filková et al., 2014; Lunavat et al., 2015
hsa-miR-23a-3p	Gastric-, colorectal-, esophageal-, liver-, renal-, breast-, prostate-, pancreas-, lung-, laryngeal-, lung cancer, CML, AML, Burkitt lymhoma, melanoma, osteosarcoma and endometriosis; retinal degeneration; UV damage; apoptosis induction; autophagy; progeria; osteoarthritis; obesity	Wang et al., 2014; Yang et al., 2014; Zheng et al., 2014
hsa-miR-451a	Lung-, colorectal-, breast-, skin-, bladder-, gastric-, renal-, esophageal-, thyroid-, liver cancer, T-ALL, AML, CML, multiple myeloma, endometriosis, prolactinoma, osteosarcoma and glioblastoma; drug transporters; cell cycle; metastasis formation; angiogenesis; rheumatoid arthritis; cardiomyopathy	Lopotová et al., 2011; Song et al., 2014; Riquelme et al., 2016

## Discussion

Regular exercise has a beneficial role in preventing a number of chronic diseases. This is primarily due to the fact that regular exercise acts at a systemic level (Anderson and Durstine, 2019). However, a gap remains between identifying in detail the molecular mechanisms induced by exercise and the observed potential benefits in health (Sanford et al., 2020). A better understanding of these biological processes and pathways could allow for the development of targeted exercise intervention and also provide basis for developing exercise-mimetic molecular level interventions (Sanford et al., 2020).

Therefore, in the present study we examined, for the first time, the effect of short-term (0.5 year in this study) and long-term (25 + years in this study) regular exercise on global circulating exomiR profile. To the best of our knowledge, this is the first study to use an amplification-free platform (Nanostring) to determine the miRNA expression profile of exercise-derived exosomes as most studies of the field evaluate specific miRNA species by amplification-based RT-qPCR (Estébanez et al., 2021). The technology applied in the current study is not only amplification-free, but also a sensitive, robust and reproducible state-of-the-art method (Hong et al., 2021). Exosomal miRNA analysis showed a significant number of differentially expressed exosomal miRNAs in all group comparisons. Comparing the miRNAs enriched or depleted in both groups (0.5 year and 25 + years), we have identified 12 similarly regulated exomiRs in the young and senior trained groups as compared to the sedentary group as shown by Figure 3 (for details please refer to Supplementary Material). The KEGG pathway analysis of similarly expressed serum-derived exomiRs confirmed their involvement in pathways related to cancer development affecting TGF-beta, p53 and mTOR signaling. In support of our observations, physical activity has been shown to be associated with lower cancer risks (Li et al., 2020). Moreover, the overall cancer incidence is lower in athletes than in the general population (Sormunen et al., 2014). Recently, a number of studies have indicated that certain exosomal miRNA species (Table 2), can be used as biomarkers of cancer and other chronic diseases (references of the studies are listed in Table 2).

An elevated expression level of miR-23a has been identified in the serum of various types of human cancer, including breast, gastric, pancreatic, and esophageal squamous cell carcinoma (Wang et al., 2019). Further analysis showed that miR-23a travels as exosomal cargo, and circulating exosomal miR-23a is up-regulated in the serum of early stage colorectal cancer patients (Yong et al., 2013). As a robust cellular regulator of gene expression, miR-23a targets a broad range of mRNA species in cancer cells by directly binding to their three prime untranslated regions (3’-UTR), which in turn suppresses gene expression (Wang et al., 2019). For example, the up-regulation of miR-23a in gastric cancer promotes cell proliferation and inhibits apoptosis (Hua et al., 2018). Zhu et al. (2010) suggested that miR-23a can target IL6R in gastric adenocarcinoma thus encouraging the proliferation of tumor cells. Based on literature data, the inhibition of miR-23a by antisense oligonucleotide inhibits proliferation and promotes the apoptosis of gastric adenocarcinoma cells (Liu et al., 2014). Its biological functions encompass drug resistance, metastasis formation and cancer progression, suggesting its potential role as an emerging targetable entity in cancer treatment (Wang et al., 2019). Of note, miR23a shows natural correlation with age, partly explaining the correlation of the above cancers with senior age (Rani et al., 2017).

Exosomal miR-451a was highly expressed in non-small-cell lung carcinoma patients (NSCLC) compared to healthy individuals. This miRNA was strongly associated with tumor progression, recurrence, and poor prognosis in NSCLC patients. According to literature data, it may serve as a potential predictive biomarker for NSCLC (Kanaoka et al., 2018). Zhu et al. (2014) also found that miR-451 levels were consistently elevated in the plasma of patients with gastric cancer providing high diagnostic accuracy for early stage gastric adenocarcinoma. To date, numerous genes have been confirmed as actual targets of miR-451, covering multiple biological signaling pathways including apoptosis, cell invasion and migration, cell proliferation and angiogenesis. Taken together, an accumulating body of evidence indicates that miR-451 is a potential biomarker for cancer diagnosis and prognosis, possibly a treatment target in combination with established drugs (Bai and Wu, 2019).

Exosomal miR-223-3p level in the serum of patients with breast cancer was significantly higher in comparison with healthy controls (Yoshikawa et al., 2018). Its expression was tightly associated with the malignancy of breast cancer, suggesting that exosomal miR-223-3p might be a useful biomarker for the early detection of invasive breast cancer. Of further note, miR223-3p also shows correlation with the advance of age, and these cancers are known to emerge at senior age (Rani et al., 2017).

Elevated expression of miR-150-5p has been shown in breast cancer (BC), described as a good prognostic biomarker for patients with HER2-positive BC (Ozawa et al., 2020).

Exercise was shown to modulate the expression of several miRNA species that in turn are protective against cancer (Li et al., 2020; Pulliero et al., 2020). In our report we demonstrate this modulation observed after both short-term (0.5 year) and long-term (25 + years) regular exercise since our miR-23a, 451a, 223-3p, and miR-150-5p were all suppressed emphasizing the role of exercise in the prevention of several cancer entities. Nevertheless, data on exercise-derived exosomal miRNA species in modulating cancer prevention is still in its infancy (Pulliero et al., 2020). Therefore, elaborate research effort is required to reveal the role of exosomal miRNA species in this particular field.

The deregulation of miRNA species described in conjunction with other chronic diseases has also been observed in our study. According to our results, regular exercise altered the levels of miR-15a and miR-142 in the opposite direction as observed in patients with diabetes and neurodegenerative disease, supporting that regular exercise (either short- or long-term) reduces the risk of developing such chronic diseases. In more detail, miR-15a was shown to be elevated in the plasma of diabetic patients also showing correlation with disease severity (Kamalden et al., 2017). Xiong et al. (2020) demonstrated that miR-15a-3p is up-regulated in exosomes of diabetic patients, and impairs wound healing. When miR-15a-3p was knocked down and such exosomes were utilized later on, their negative effects on the metabolism and wound healing in particular were partially reversed both *in vitro* and *in vivo* (Xiong et al., 2020).

Barbagallo et al. (2020) isolated exosomal miRNA from the serum of 30 Parkinson disease (PD) patients and compared it with that of 30 healthy controls. The expression levels of ex-miR-23a; ex-miR-142-3p were significantly elevated in the serum of PD patients, unlike in our study where miR-142 showed a decrease in expression compared to healthy, but sedentary state (Barbagallo et al., 2020). Previous studies have also reported the benefits of physical exercise in improving the symptoms in individuals with PD (Da Silva et al., 2021). Taken together these reports suggest the protective role of miR-142-3p in PD, though further studies are required.

Taken together, the miRNAs that we have observed to be modulated by both short and long-term exercise are mostly involved in cancer prevention mechanisms including tumor suppression (miR-223-3p; miR-451a; miR-15a/b-5p; let-7a/7g-5p) (Wang et al., 2016; Gao et al., 2011), aging (miR-223-3p; miR-451a; miR-15a/b-5p; miR-23a-3p) (Mercken et al., 2013; Teteloshvili et al., 2015), induction of apoptosis (miR-150-5p; miR-15a/b-5p; miR-130a-3p) (Xu et al., 2014; Wang et al., 2015) and reduction of inflammation (miR-199a/b-3p; miR-142-3p) (Cai et al., 2012). In addition, the inverse deregulation of miR-15a characteristic to diabetes, and miR-142 featured in Parkinson’s disease has also been recorded in our study. Potential applications target these miRNA species to prevent the development of cancer, diabetes and neurodegenerative disease or to be used as adjuvant therapy in established diseases. However, to date no such experiments exist supporting that exercise-derived exomiRs could prevent or treat chronic diseases.

So, beyond the utility of serum-derived exomiRs as potential biomarkers of physical fitness or chronic diseases, our work suggests their key role in essential pathways, potentially preventing the development of multiple chronic diseases. In the future the evaluation of physical activity level may be used to predict the risk of developing various chronic diseases. Furthermore, this study is important as a starting point to understand the global pattern of regular exercise-related exomiRs and their target pathways in health and disease. However, the present study must be seen as an exploratory study. Our current pilot-study is limited by the number of biological replicates. Therefore, further studies are required with larger sample size to comprehensively examine the effect of regular exercise on circulating exomiR profile.

## Conclusion

Both short- (0.5 year) and long-term (25 + years) regular exercise significantly alters the serum miRNA profile in healthy individuals, potentially reducing the risk of a number of malignant, metabolic and neurodegenerative diseases. Combining an amplification-free miRNome profiling platform and bioinformatics analysis, our study revealed that numerous disease-associated exomiRs show differential expression toward a more beneficial pattern. Physiological relevance is also supported by the large number of genes targeted by these miRNAs. Future work lies ahead in determining the exact mechanism of action and the potential use of exomiRs as therapeutic tools to efficiently prevent or successfully treat age-related diseases.

## Data Availability Statement

The datasets presented in this study can be found in online repositories. The names of the repository/repositories and accession number(s) can be found below: EBI ArrayExpress, accession no: E-MTAB-10067.

## Ethics Statement

The studies involving human participants were reviewed and approved by the Regional and Local Ethics Committee of Clinical Centre, University of Pecs (ref. no.: 6439/2016 and 7755/2019). The patients/participants provided their written informed consent to participate in this study.

## Author Contributions

KG, KK, and MW designed the study. KG, ZA, KB, and AdG recruited participants, collected samples and performed the experiments. RH and AtG analyzed the data. KG and ZA interpreted data and drafted the manuscript. JP, MW, and KK critically revised the manuscript. All authors contributed to the article and approved the submitted version.

## Conflict of Interest

The authors declare that the research was conducted in the absence of any commercial or financial relationships that could be construed as a potential conflict of interest.

## Publisher’s Note

All claims expressed in this article are solely those of the authors and do not necessarily represent those of their affiliated organizations, or those of the publisher, the editors and the reviewers. Any product that may be evaluated in this article, or claim that may be made by its manufacturer, is not guaranteed or endorsed by the publisher.

## Footnotes

1
http://bioinfo.life.hust.edu.cn/EVmiRNA
