# Corrigendum: Physical Activity as a Preventive Lifestyle Intervention Acts Through Specific Exosomal miRNA Species—Evidence From Human Short- and Long-Term Pilot Studies

**DOI:** 10.3389/fphys.2021.794940

**Published:** 2021-11-22

**Authors:** Kitti Garai, Zoltan Adam, Robert Herczeg, Krisztina Banfai, Adam Gyebrovszki, Attila Gyenesei, Judit E. Pongracz, Marta Wilhelm, Krisztian Kvell

**Affiliations:** ^1^Department of Pharmaceutical Biotechnology, Faculty of Pharmacy, University of Pécs, Pécs, Hungary; ^2^Wnt-Signaling Research Group, Szentagothai Research Center, University of Pécs, Pécs, Hungary; ^3^Bioinformatics Research Group, Szentagothai Research Center, University of Pécs, Pécs, Hungary; ^4^Faculty of Science, Institute of Sport Sciences and Physical Education, University of Pécs, Pécs, Hungary

**Keywords:** regular exercise, exosome, miRNA, chronic disease, prevention

In the original article, there was a mistake in the Funding statement. We mistakingly included the funder “TUDFO/51757-1/2019-ITM”, the correct funder is “2020-4.1.1/TKP2020 Biomedical Engineering”.

The authors apologize for this error and state that this does not change the scientific conclusions of the article in any way. The original article has been updated.

## Publisher's Note

All claims expressed in this article are solely those of the authors and do not necessarily represent those of their affiliated organizations, or those of the publisher, the editors and the reviewers. Any product that may be evaluated in this article, or claim that may be made by its manufacturer, is not guaranteed or endorsed by the publisher.

