# Supplementary material for: Physical Activity as a Preventive Lifestyle Intervention Acts Through Specific Exosomal miRNA Species—Evidence From Human Short- and Long-Term Pilot Studies
Source: Front Physiol. 2021 Aug 2;12:658218. doi: 10.3389/fphys.2021.658218 (PMC8365358; doi:10.3389/fphys.2021.658218)
Supplement: Supplementary file 1 [file Table_1.DOCX]

| **Table 1.** | | | | |
| --- | --- | --- | --- | --- |
| Subject characteristics | | | | |
|  | **Baseline** | **0.5 years** | ***p*** | **25+ years** |
|  |  |  |  |  |
| **Age (years)** | 23 ± 2 | 23.5 ± 2 |  | 62 ± 6 |
| **BMI** | 21.64 ± 1.57 | 21.46 ± 1.44 | 0.382 | 27.92 ± 2.95 |
| **Body Weight (kg)** | 60.39 ± 5.42 | 59.55 ± 5.74 | 0.166 | 75.16 ± 7.18 |
| **Body Fat Percentage (%)** | 31.79 ± 3.39 | 31.49 ± 3.47 | 0.61 | 21.23 ± 6.03 |
| **VO_2_ max (ml/kg/min)** | 36.41 ± 6.67 | 39.81 ± 6.20* | 0.047 | 32.9 ± 6.99 |
| **LDL (mmol/L)** | 2.35 ± 0.9 | 2.44 ± 0.83 | 0.481 | 3.63 ± 1.19 |
| **HDL (mmol/L)** | 1.81 ± 0.55 | 2.13 ± 0.61* | 0.002 | 1.68 ± 0.53 |
| **Glucose (mmol/L)** | 4.94 ± 0.39 | 4.63 ± 0.31** | <0.001 | 5.67 ± 0.45 |
| **Systolic BP (Hgmm)** | 114.5 ± 14.18 | 108.07 ± 8.69 | 0.55 | 131.2 ± 19.42 |
| **Diastolic BP (Hgmm)** | 76.07 ± 9.19 | 72.93 ± 7.92 | 0.223 | 87.00 ± 9.25 |
| Values are expressed as mean +/− SD. Paired t-test (*, p<0.05; **, p<0.001); p values were calculated for baseline vs. 0.5 year as applicable; VO_2_ max, maximal oxygen uptake (cardiorespiratory fitness); LDL, Low Density Lipoprotein; HDL, High Density Lipoprotein; BP, Blood Pressure | | | | |
