# Supplementary material for: Physical Activity as a Preventive Lifestyle Intervention Acts Through Specific Exosomal miRNA Species—Evidence From Human Short- and Long-Term Pilot Studies
Source: Front Physiol. 2021 Aug 2;12:658218. doi: 10.3389/fphys.2021.658218 (PMC8365358; doi:10.3389/fphys.2021.658218)
Supplement: Supplementary file 2 [file Table_2.DOCX]

**Table 2: Summary of** **exomiR biomarkers related to certain diseases according to EVmiRNA database**

| **miRNA species** | **Affected age-related chronic disease, autoimmune condition or infection** | **References** |
| --- | --- | --- |
| hsa-let-7a-5p | Colorectal-, renal-, prostate-, ovarian-, breast-, lung-, pancreas-, gastric-, esophageal-, thyriod cancer, Ewing's and Kaposi's sarcoma, glioblastoma, AML and MML; metastasis formation; cell cycle control; inflammation; diabetes; cardiovascular disease; hepatitis B infection | (Trang et al. 2010);  (Lee et al. 2011);  (Iliopoulos et al. 2009) |
| hsa-let-7g-5p | Breast-, esophageal-, lung cancer, glioblastoma, AML and CML; graft-versus-host disease; inflammation; autoimmune thyroid disease; cell cycle control; diabetes; cardiovascular disease; metabolic syndrome; hepatitis B and influenza A infection | (Arora et al. 2011);  (Biamonte et al. 2019);  (Wang et al. 2013) |
| hsa-miR-130a-3p | Lung-, liver-, prostate-, ovarian-, breast-, cervical-, nasopharyngeal-, prostate cancer, myeloma, CML and glioblastoma; cardiovascular disease; fibrosis; inflammation; autophagy; diabetes; Crohn's disease; hepatitis C infection; cardiac arrhythmia; renal GBM disease; UV damage | (Eichelmann et al. 2018);  (Osbourne et al. 2014);  (Huang et al. 2015) |
| hsa-miR-142-3p | Liver-, lung-, colorectal-, breast-, cervical-, esophageal cancer, osteosarcoma, prolactinoma, ALL, AML, CLL and MALT lymphoma; graft rejection; Hashimoto's thyroiditis; multiple sclerosis; cardiovascular disease; inflammation; rotavirus infection; Alzheimer's disease; fibrosis | (Wang et al. 2017);  (Sukma Dewi et al. 2017);  (Ma et al. 2016) |
| hsa-miR-150-5p | Colorectal-, lung-, liver-, prostate-, cervical-, pancreas-, breast-, ovarian-, esophageal cancer, osteosarcoma, glioblastoma, melanoma; Burkitt lymphoma, ALL and MML; inflammation; cardiovascular disease; fibrosis; irritable bowel syndrome; myasthenia; diabetes; SLE; psoriasis | (Qu et al. 2014);  (Roderburg et al. 2013);  (Yu et al. 2015) |
| hsa-miR-15a-5p | Gastric-, colorectal-, lung-, breast-, liver-, ovarian-, prostate cancer, melanoma, osteosarcoma, neuroblastoma, pheochromocytoma, AML, CLL and multiple myeloma; inflammation; cell cycle control; apoptosis induction; autophagy; multiple sclerosis; hepatitis B infection; fibrosis; diabetes | (Xia et al. 2008);  (Bandi et al. 2009);  (Sun et al. 2013) |
| hsa-miR-15b-5p | Liver-, gastric-, lung-, liver-, pancreas-, ovarian-, squamous cell cancer, glioblastoma, melanoma, CLL and thymoma; apoptosis induction; metastasis formation; angiogenesis; fibrosis; bipolar disorder; insulin-resistance; skin photoaging; multiple sclerosis; diabetes | (Li et al. 2016);  (MacLean et al. 2016);  (Zhang et al. 2015b) |
| hsa-miR-199a/b-3p | Liver-, gastric-, lung-, renal cell-, ovarian-, pancreas-, colorectal, liver-, breast-, testicular germ cell-, thyroid-, colorectal cancer, endometriosis, glioblastoma, CLL, melanoma, chondrosarcoma and osteosarcoma; osteoarthritis; COPD; autophagy; angiogenesis; HCV infection; inflammation | (Li et al. 2015) |
| hsa-miR-223-3p | Ovarian-, gastric-, colorectal-, prostate-, pancreas-, lung-, liver cancer, CLL, AML, ALL, glioblastoma and osteosarcoma; metastasis formation graft rejection; inflammation; osteoarthritis; lipid metabolism; obesity; rheumatoid arthritis; psoriasis; cardiovascular disease; diabetes; COPD; Alzheimer's disease | (Lunavat et al. 2015);  (Wong et al. 2008);  (Filková et al. 2014)v |
| hsa-miR-23a-3p | Gastric-, colorectal-, esophageal-, liver-, renal-, breast-, prostate-, pancreas-, lung-, laryngeal-, lung cancer, CML, AML, Burkitt lymhoma, melanoma, osteosarcoma and endometriosis; retinal degeneration; UV damage; apoptosis induction; autophagy; progeria; osteoarthritis; obesity | (Zheng et al. 2014);  (Wang et al. 2014);  (Yang et al. 2014) |
| hsa-miR-451a | Lung-, colorectal-, breast-, skin-, bladder-, gastric-, renal-, esophageal-, thyroid-, liver cancer, T-ALL , AML, CML, multiple myeloma, endometriosis, prolactinoma, osteosarcoma and glioblastoma; drug transporters; cell cycle; metastasis formation; angiogenesis; rheumatoid arthritis; cardiomyopathy | (Lopotová et al. 2011);  (Riquelme et al. 2016);  (Song et al. 2014) |
